# Supplementary material for: Effects of Environmental Concentrations of Total Phosphorus on the Plankton Community Structure and Function in a Microcosm Study
Source: Int J Environ Res Public Health. 2022 Jul 9;19(14):8412. doi: 10.3390/ijerph19148412 (PMC9324525; doi:10.3390/ijerph19148412)
Supplement: Supplementary file 1 [file ijerph-19-08412-s001.zip › ijerph-1750086-supplementary.pdf]

Supplementary Information

Effects of environmental concentrations of total phosphorus on the plankton community structure and function in a microcosm study

Xue Bai, Zhendong Jiang, Yuan Fang, Lin Zhu, Jianfeng Feng*

Key Laboratory of Pollution Process and Environmental Criteria of Ministry of Education and Tianjin Key Laboratory of Environmental Remediation and Pollution Control, College of Environmental Science and Engineering, Nankai University, Tianjin, 300071, P. R. China

Table S1 Algae Group Index (AGI) of the phytoplankton community under different total phosphorus (TP) concentrations.

Treatment	Algae Group Index (AGI)							
	7 d	14 d	21 d	28 d	35 d	42 d	49 d	56 d
Control	5.20 ± 1.12	2.35 ± 0.20	5.33 ± 1.91	5.61 ± 1.53	4.17 ± 1.62	1.14 ± 0.21	2.27 ± 0.13	4.67 ± 1.16
TP 0.10 mg/L	14.96 ± 3.02 *	4.72 ± 1.25	3.20 ± 0.85	7.33 ± 3.19	18.04 ± 4.16 *	2.19 ± 0.47	2.45 ± 0.47	2.62 ± 0.38
TP 0.20 mg/L	14.75 ± 7.85	7.73 ± 2.32	2.72 ± 0.84	3.88 ± 0.80	5.80 ± 0.81	7.84 ± 3.82	2.19 ± 0.26	3.65 ± 0.33
TP 0.30 mg/L	9.50 ± 0.13 *	8.29 ± 2.75	7.20 ± 2.12	4.12 ± 1.14	6.62 ± 2.19	7.26 ± 2.50	4.56 ± 1.51	8.01 ± 3.36
TP 0.40 mg/L	9.55 ± 2.17	5.19 ± 2.15	11.25 ± 4.12	4.95 ± 1.02	9.74 ± 0.40 *	15.42 ± 4.71	2.78 ± 0.45	7.76 ± 3.27

Note: $p \leq 0.05$ is marked as *, $p \leq 0.01$ is marked as **, and $p \leq 0.001$ is marked as ***.

Table S2 Percentages of total variance attributed to time and treatment regime in all principal response curve (PRC) analyses performed in this study. The table also indicates which fraction of the variance explained by treatment regime is captured by first PRC.

Dataset	% Variance accounted for by			% Variance explained by treatment regime (first PRC)
	Time	Treatment	Residual	
Phytoplankton	24.76%	36.57%	38.67%	26.47%
Zooplankton	52.54%	28.44%	19.02%	33.52%

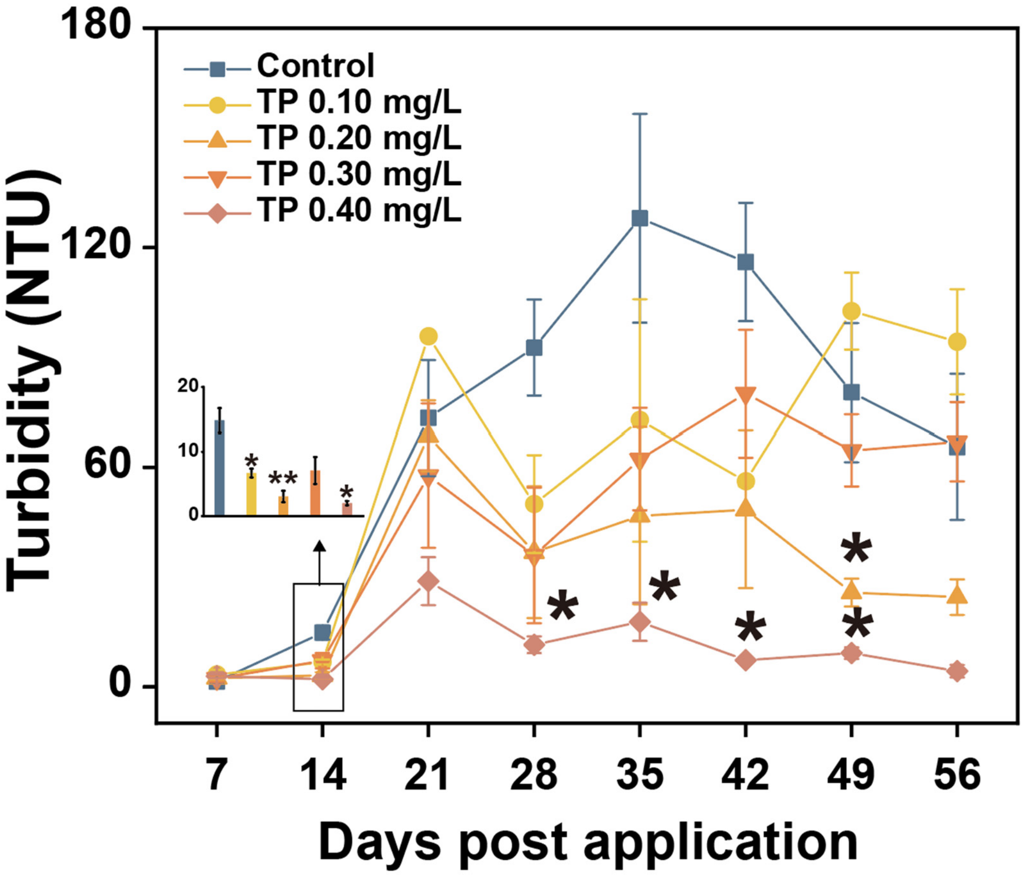

Figure S1. Changes in turbidity under different total phosphorus (TP) concentrations. The bar chart indicated by the arrow represents the changes of turbidity under different TP treatments on day 14.

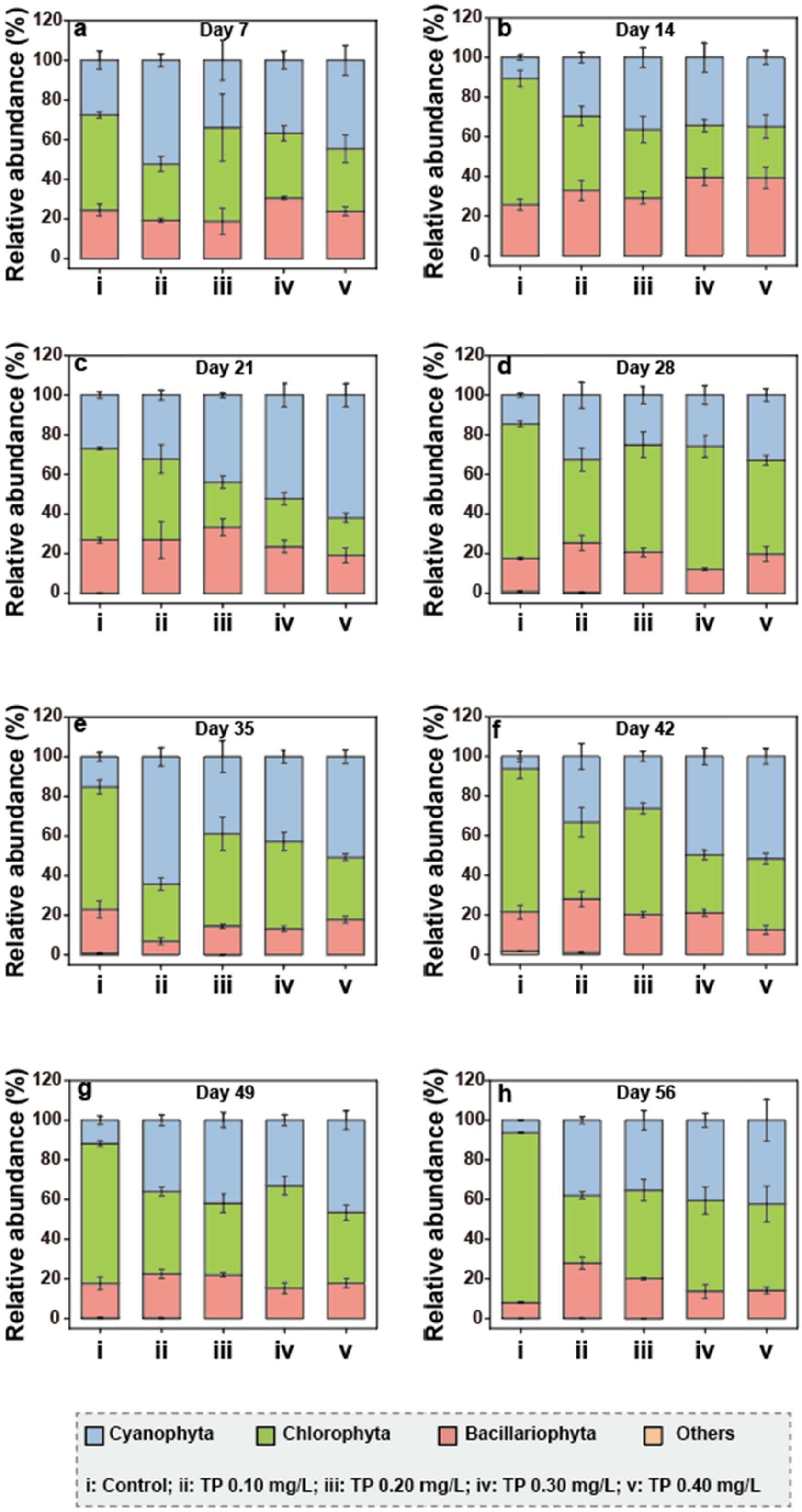

Figure S2. Relative abundances of phytoplankton taxa groups under different TP concentrations.

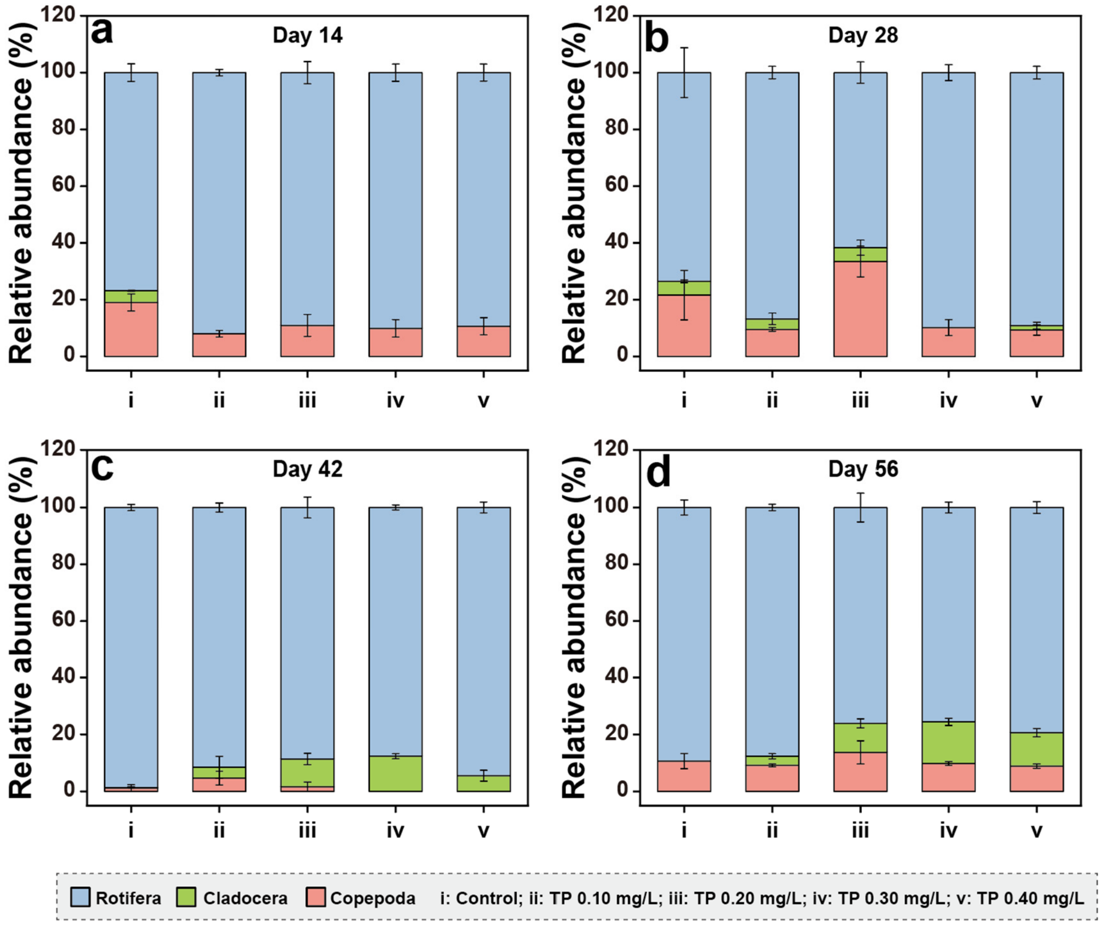

Figure S3. Relative abundances of zooplankton taxa groups under different TP concentrations.
